# Profile of sleep pattern, psychiatric comorbidity and problematic electronic gadget use in children and adolescents with autism and ADHD

**DOI:** 10.1192/bjo.2021.143

**Published:** 2021-06-18

**Authors:** Darpan Kaur, Sanay Patani, Rishab Verma, Rakesh Ghildiyal

**Affiliations:** 1 Department of Psychiatry, Mahatma Gandhi Missions Medical College and Hospital, Sector 01, Kamothe, Navi Mumbai, Maharashtra, India; 2 Mahatma Gandhi Missions Medical College and Hospital, Sector 01, Kamothe, Navi Mumbai, Maharashtra, India

## Abstract

**Aims:**

To assess the profile of Sleep pattern, Psychiatric comorbidity and problematic electronic gadget use and explore demographic factors and correlations in children and adolescents with ADHD and Autism.

Hypothesis: There are statistically significant problems and associations across sleep pattern, psychiatric comorbidity and gadget use in children and adolescents with autism and ADHD.

**Background:**

Literature highlights increasing global trends and emerging concerns over the problematic use of electronic gadgets and sleep related problems in children and adolescents with autism and ADHD. There is sparse literature on the profile of sleep patterns, psychiatry comorbidity and problematic gadget use in children and adolescents with autism and ADHD from developing countries.

**Method:**

This was an observational study conducted at the Child and Adolescent Psychiatry Clinic, Department of Psychiatry at a tertiary care Institution under the STS ICMR Project 2019 with Institutional Ethics Clearance. Apriori Sample size calculated was 70. Children and adolescents diagnosed with autism or ADHD as per ICD 10 criteria, fulfilling the inclusion criteria and willing to participate in the study were included. Informed consent was obtained from caregivers. Sleep Disturbance Scale for Children, Self Designed Parent based Problematic Electronic Gadget Use Scale, Vanderbilt ADHD scale, Indian scale for Assessment of Autism and the Child and Adolescent Psychiatry Clinic structured Performa were the tools for data collection. The results were analyzed with descriptive tests, chi square test and multiple logistic regressions using SPSS.

**Result:**

Mean age of the sample was 9.1 years and majority (57%) were boys. Forty nine patients had ADHD and 21 patients had Autism. Problematic gadget use was higher in children ranging from 6 to 15 years of age and 12.8% had severe levels of problematic gadget use. 34.3% patients experienced severe problems in initiating and maintaining sleep. Oppositional-Defiant disorder was the most common comorbidity, predominantly inattentive type (76.4%) was the most common subtype of ADHD and mild autism (54.3%) was the most common type of autism in the sample. There were statistically significant associations (p < 0.05) between age and gadget use; hyperactive subtype of ADHD and problems with initiating and maintaining sleep and ADHD subtype, sex profile and problematic gadget use.

**Conclusion:**

We conclude that sleep problems, psychiatric comorbidity and problematic gadget use are prevalent with statistically significant associations in children and adolescents with autism and ADHD as per our study findings. Our study has relevant clinical, research and policy implications.

